# Supplementary material for: Active site specificity profiling datasets of matrix metalloproteinases (MMPs) 1, 2, 3, 7, 8, 9, 12, 13 and 14
Source: Data Brief. 2016 Feb 22;7:299–310. doi: 10.1016/j.dib.2016.02.036 (PMC4777984; doi:10.1016/j.dib.2016.02.036)
Supplement: Supplementary file 10 — Supplementary material [file mmc10.zip › WebPICS_hMMP14_T_1%/P2prime.html]

 

PICS results


|  |  |
| --- | --- |
| **P2prime\_C**  6 in 307 sites   2.0 %    effects > 10 perc. pnts.  (vice-versa in brackets)  P3prime\_Q: 45.8 (21.1) |  |
| **P2prime\_H**  13 in 307 sites   4.2 %    effects > 10 perc. pnts.  (vice-versa in brackets)  P1prime\_W: 11.5 (12.5) |  |
| **P2prime\_I**  35 in 307 sites   11.4 %    effects > 10 perc. pnts.  (vice-versa in brackets)  P3\_L: 13.1 (15.3)   P2\_Q: 16.1 (26.7) |  |
| **P2prime\_L**  49 in 307 sites   16.0 %    effects > 10 perc. pnts.  (vice-versa in brackets)  P2\_A: -15.4 (-11.5) |  |
| **P2prime\_M**  15 in 307 sites   4.9 %    effects > 10 perc. pnts.  (vice-versa in brackets)  P3\_H: 10.7 (20.1)   P3prime\_V: 18.2 (10.5) |  |
| **P2prime\_Q**  25 in 307 sites   8.1 %    effects > 10 perc. pnts.  (vice-versa in brackets)  P1prime\_Q: 15.4 (27.6) |  |
| **P2prime\_W**  5 in 307 sites   1.6 %    effects > 10 perc. pnts.  (vice-versa in brackets)  P1\_M: 17.4 (10.9) |  |
